# Identifying Long‐Term Trajectories of Foot Pain Severity and Potential Prognostic Factors: A Population‐Based Cohort Study

**DOI:** 10.1002/acr.24823

**Published:** 2022-11-25

**Authors:** Michelle Marshall, Milica Blagojevic‐Bucknall, Trishna Rathod‐Mistry, Martin J. Thomas, John J. Edwards, George Peat, Hylton B. Menz, Edward Roddy

**Affiliations:** ^1^ Primary Care Centre Versus Arthritis, School of Medicine Keele University Staffordshire UK; ^2^ Primary Care Centre Versus Arthritis, School of Medicine, Keele University, Staffordshire, UK, and Haywood Academic Rheumatology Centre, Midlands Partnership NHS Foundation Trust, Haywood Hospital Burslem Staffordshire UK; ^3^ Primary Care Centre Versus Arthritis, School of Medicine, Keele University, Staffordshire, UK, and School of Allied Health, Human Services and Sport, College of Science, Health and Engineering, La Trobe University Melbourne Victoria Australia

## Abstract

**Objectives:**

To identify distinct foot pain trajectories over 7 years and examine their associations with potential prognostic factors.

**Methods:**

Adults ages ≥50 years and registered with 4 general practices in North Staffordshire, UK were mailed a baseline health survey. Those reporting current or recent foot pain were invited to attend a research assessment clinic. Follow‐up was by repeated postal surveys at 18, 36, 54, and 84 months. Distinct trajectories of foot pain were explored using latent class growth analysis (LCGA). Subsequently, identified trajectories were combined into most and least progressive groups, and covariate‐adjusted associations with a range of prognostic factors were examined.

**Results:**

Of 560 adults with foot pain attending baseline research clinics, 425 (76%) provided data at baseline and 2 or more follow‐up time points. LCGA for foot pain severity (0–10 numerical rating scale) identified a 4‐trajectory model: “mild, improving” (37%); “moderate, improving” (33%); “moderate‐severe, persistent” (24%); and “severe, persistent” (6%). Compared with individuals in more favorable (improving) pain trajectories, those in less favorable (persistent) pain trajectories were more likely to be obese, have routine/manual and intermediate occupations, have poorer physical and mental health, have catastrophizing beliefs, have greater foot‐specific functional limitation, and have self‐assessed hallux valgus at baseline.

**Conclusions:**

Four distinct trajectories of foot pain were identified over a 7‐year period, with one‐third of individuals classified as having pain that is persistently moderate‐severe and severe in intensity. The effect of intervening to target modifiable prognostic factors such as obesity and hallux valgus on long‐term outcomes in people with foot pain requires investigation.

## INTRODUCTION

Foot pain affects 24% of adults ages ≥45 years and accounts for 8% of primary care musculoskeletal consultations in the UK (1, 2). Painful foot disorders are major causes of restricted activity, poor balance, and risk of falling, particularly in older people (1, 3). Symptomatic osteoarthritis (OA) is the leading global cause of years lived with disability in older people (4) and is likely to be a significant contributor to foot pain in this age group (5).

**SIGNIFICANCE & INNOVATIONS**

- Individuals with joint pain often believe their pain will get worse over time, but there are few prospective studies of foot pain and osteoarthritis (OA).
- Over 7 years, one‐third of individuals had persistent moderate‐severe or severe foot pain. Reassurance is provided that many people with foot pain improve over time.
- Persistent pain was associated with obesity, routine/manual and intermediate occupations, poorer physical and mental health, catastrophizing beliefs, foot‐specific functional limitation, and hallux valgus at baseline.
- Future research should investigate whether targeting modifiable prognostic factors, such as obesity and hallux valgus, improves long‐term outcomes in people with foot pain.

Few prospective studies of foot pain and OA have been undertaken, and little is known about what happens to people over time. Longitudinal studies of foot OA to date have focused on radiographic progression rather than change in symptoms, finding that structural progression after 3 and 19 years occurred in only one‐third of people (6, 7). At other joint sites, including the knee, hip, and hand, distinct pain trajectories and specific generic and joint‐specific risk factors for pain progression have been identified, showing that OA symptoms fluctuate over time and vary greatly between individuals (8, 9, 10, 11, 12).

Better understanding of the prognosis and natural history of foot pain is needed to develop a coherent clinical approach to helping people with painful foot OA and strategies to reduce the impact of related pain and disability in older people. This information could also help identify where treatment need is greatest, whether there are modifiable risk factors that could be targeted, and to inform intervention development for future clinical trials. The objectives of this study were to identify distinct trajectories of foot pain and function over 7 years in community‐dwelling individuals ages ≥50 years and examine associations between trajectories and potential prognostic factors.

## PATIENTS AND METHODS

### Study population and data collection

This was a prospective observational cohort study, the Clinical Assessment Study of the Foot (CASF) (13). Ethical approval was obtained at baseline, 18‐, 36‐, and 54‐month follow‐up from Coventry Research Ethics Committee (REC project number: 10/H1210/5) and at 84‐month follow‐up from East Midlands—Leicester South Research Ethics Committee (REC project number: 18/EM/0249).

At baseline (May 2010 to July 2011), a postal health survey was sent to community‐dwelling adults ages ≥50 years registered with 4 general practices in North Staffordshire, UK. The health survey collected information on foot pain severity (0–10 numerical rating scale [NRS]) (14); foot pain and foot‐specific function (Manchester Foot Pain and Disability Index [MFPDI]) (15); self‐reported hallux valgus using a validated line drawing instrument for which the 3 most severe grades (representing 30, 45, and 60 degrees of angulation) indicate presence of the condition (16); general health (Short Form 12 health survey [SF‐12]) (17); anxiety and depression (Hospital Anxiety and Depression Scale [HADS]) (18); coping strategies (19); and demographic and socioeconomic characteristics. Participants provided written informed consent to participate and to further contact in the postal questionnaire.

Individuals who reported experiencing foot pain within the preceding 12 months at baseline and provided consent to further contact were invited to attend a research clinic where assessments were undertaken, including the Foot Posture Index (FPI) (20), and height and weight were measured. Weight‐bearing dorsoplantar and lateral radiographs of each foot were obtained. Osteophytes and joint space narrowing were graded separately in the first metatarsophalangeal (MTP) joint, first and second cuneometatarsal joints, navicular‐first cuneiform joint, and talonavicular joint using a 0–3 scale according to a standardized foot atlas (21). Radiographic OA was defined as grade ≥2 for either feature on either view in 1 or more joints. Symptomatic radiographic foot OA phenotypes (no/minimal foot OA, isolated first MTP joint OA, polyarticular foot OA) were defined as described previously (22). Further written informed consent was obtained to undergo assessment at the research clinic.

Participants’ consent for review of their medical records was requested. Participants with gout were identified from the primary care medical records, and inflammatory arthritis (nonspecific inflammatory arthritis, rheumatoid arthritis, or psoriatic arthritis) were identified from the primary care and hospital medical records and a clinical radiography report by a consultant musculoskeletal radiologist.

All baseline, clinic attenders received follow‐up postal health surveys at 18, 36, 54, and 84 months, except deceased participants and those who had revoked consent or whose general practitioner had deemed them inappropriate to contact. At each time point, survey content was similar to baseline and, nonresponders were sent reminders 2 and 4 weeks after mailing, followed by attempts to collect key outcomes (MFPDI function subscale, global assessment of change, and pain, aching, and stiffness in previous month) by telephone and mail.

### Outcomes

The main outcome was foot pain severity in the previous month (0–10 NRS) completed at each of the 5 time points (14). The impact of foot pain and function were also assessed at all time points using the MFPDI pain and functional limitation subscales (15). These subscales fit the Rasch model, allowing interval‐level data to be generated. Higher Rasch‐transformed scores indicate greater pain and limitation (23).

### Public and patient involvement

People with foot pain contributed to study design, conduct, and dissemination. They prioritized pain and impaired function as the most important outcomes and informed questionnaire content. Individuals assisted with baseline clinical assessment training, refining the assessment protocol. They contributed to interpretation of findings and dissemination plans.

### Statistical analysis

Latent class growth analysis (LCGA) was used to model repeated measures of foot pain (0–10 NRS) at 5 time points over 84 months. For inclusion in the analysis, participants were required to have foot pain data at baseline and 2 or more of the 4 follow‐up time points. The optimal number of trajectories was selected using a combination of statistical, parsimony, and interpretability criteria (24).

Three models were tested initially, with solely linear, quadratic, or cubic specifications for trajectories. In each case, the number of modeled trajectories was increased and significance of the improvement in the model fit assessed using Akaike information criterion (AIC), Bayesian information criterion (BIC), and sample size–adjusted (SSA) BIC (smaller values indicating better fit). The Lo‐Mendell‐Rubin adjusted likelihood ratio test (LRT) (25) and the bootstrap LRT were used to assess whether there was a significant improvement in model fit between K‐1 and K trajectory models. Additionally, the following criteria were used for model selection: 1) delineation of trajectories assessed by higher entropy (range 0–1), 2) clear classification of participants into trajectories (average posterior probability >0.7) (26), 3) trajectory membership ≥4% of the study population, and 4) clinical relevance and interpretation of the identified trajectories (24). Mixed polynomial models were derived, allowing the specification for trajectories to differ. An appropriate polynomial functional form for each trajectory was chosen, based on the significance of the estimated parameters related to each polynomial component (26), with each participant assigned to the most appropriate trajectory using the maximum probability assignment principle. Improvement of model fit with each additional trajectory was then assessed.

We assessed the sensitivity of our findings by modifying various components of our analyses. A complete case analysis of those with foot pain severity data at all 5 time points was undertaken to examine whether trajectories could be replicated. We also examined whether similar trajectories could be identified using the MFPDI pain and functional limitation subscales. LCGA assumes that individual participant patterns within a particular trajectory are homogeneous. We relaxed this assumption and allowed growth parameters to vary within trajectories by applying a growth mixture model (GMM), of which LCGA is a subclass. For all LCGA and GMM models, full information maximum likelihood estimation with robust standard errors was employed, and to avoid convergence at local maxima, each model was fitted using 1,000 random sets of starting values and 50 final stage optimizations carried out for each.

A nested case–control study was undertaken to compare those in the most progressive (cases) and least progressive trajectories (controls) of the final selected model. Crude univariable associations between progression and baseline characteristics, including sociodemographic (age, sex, body mass index [BMI], attended higher education, occupational class), general and mental health (SF‐12, HADS, catastrophizing beliefs), and foot‐specific characteristics (FPI, MFPDI, presence of hallux valgus, radiographic OA phenotype) (odds ratios [ORs] and 95% confidence intervals [95% CIs]) were assessed using logistic regression models and subsequently adjusted for age, sex, and BMI as potential confounders of OA severity. Additionally, the relationship between the most progressive and least progressive foot pain NRS trajectories and the MFPDI function subscale at 7 years was examined and adjusted for baseline score, age, sex, and BMI.

The Guidelines for Reporting on Latent Trajectory Studies checklist for reporting latent trajectory studies was followed (27). *P* values less than or equal to 0.05 were considered significant. Analyses were performed in STATA (version 15.0) and Mplus (version 8.0) (28).

## RESULTS

A total of 560 participants who reported having foot pain in the last 12 months and consented to further contact attended the baseline research assessment clinic. Follow‐up data were received from 501 participants (89%) at 18 months, 455 (81%) at 36 months, 356 (64%) at 54 months, and 309 (55%) at 84 months. A total of 425 participants (76%) had pain NRS data at baseline and 2 or more follow‐up time points. These participants were younger (mean age 64.4 versus 66.8 years), were less likely to be female (52.7 versus 65.9%), had significantly lower foot pain NRS (mean 5.2 versus 5.8), and had a lower SF‐36 score (mean 30.1 versus 32.0) at baseline than the remaining participants who had data at baseline but fewer than 2 follow‐up time points.

A total of 396 participants (94.3%) reported foot pain in the last month; more than half reported having foot pain, aching, or stiffness on most or all days (Table 1). Nearly two‐thirds had radiographic foot OA affecting 1 or more foot joints, 44.5% had self‐reported hallux valgus, and only 3.5% had gout and 4.0% inflammatory arthritis. Concomitant foot conditions were common (32.2%, n = 137).

**Table 1:** Baseline characteristics of the study population and the 4‐trajectory model obtained by LCGA*

Baseline characteristics	All participants (n = 425)	Mild improving pain (n = 156)	Moderate improving pain (n = 139)	Moderate‐severe persistent pain (n = 104)	Severe persistent pain (n = 26)
Age, mean ± SD years	64.4 ± 7.8	64.4 ± 7.3	64.2 ± 8.2	65.0 ± 8.1	62.4 ± 7.3
Female sex	224 (52.7)	77 (49.4)	79 (56.8)	58 (55.8)	10 (38.5)
BMI, kg/m^2^; mean ± SD	30.2 ± 5.5	29.1 ± 4.7	30.4 ± 5.8	31.1 ± 5.5	32.5 ± 6.0
Attended higher education	114 (27.6)	51 (33.6)	37 (27.6)	18 (17.8)	8 (30.8)
Occupational class:					
Managerial and professional	111 (26.1)	55 (35.3)	35 (25.2)	19 (18.3)	2 (7.7)
Intermediate	82 (19.3)	26 (16.7)	25 (18.0)	26 (25.0)	5 (19.2)
Routine and manual	210 (49.4)	71 (45.5)	71 (51.1)	52 (50.0)	16 (61.5)
SF‐12 PCS 0–100; mean ± SD	38.9 ± 12.1	44.4 ± 10.2	38.7 ± 12.3	33.4 ± 10.8	28.2 ± 8.8
SF‐12 MCS 0–100; mean ± SD	49.9 ± 10.6	53.2 ± 8.5	49.9 ± 10.6	47.0 ± 11.2	40.5 ± 10.9
SF‐36 Physical Functioning scale 0–100; mean ± SD	59.6 ± 30.1	75.7 ± 20.5	58.8 ± 30.3	44.0 ± 29.1	29.2 ± 24.0
HADS anxiety 0–21; mean ± SD	6.9 ± 4.3	5.7 ± 3.9	6.6 ± 4.0	8.1 ± 4.4	10.8 ± 4.8
HADS depression 0–21; mean ± SD	5.3 ± 3.8	3.9 ± 2.9	5.0 ± 3.5	6.6 ± 4.0	9.9 ± 4.3
Any foot pain, aching, or stiffness in last month	397 (93.4)	132 (84.6)	136 (97.8)	103 (99.0)	26 (100)
Foot pain, aching, or stiffness on most or all days in last month	234 (55.1)	54 (34.6)	77 (55.4)	78 (75.0)	25 (96.2)
Foot pain severity (NRS 0–10) last month; mean ± SD	5.2 ± 2.6	3.5 ± 2.2	5.4 ± 2.1	6.7 ± 1.9	8.6 ± 1.7
MFPDI Pain subscale (Rasch); mean ± SD	−0.21 ± 1.5	−1.18 ± 1.4	−0.10 ± 1.3	0.62 ± 1.2	1.61 ± 1.2
MFPDI Function subscale (Rasch); mean ± SD	−0.76 ± 2.1	−2.02 ± 1.6	−0.65 ± 1.9	0.42 ± 1.8	1.5 ± 1.8
FPI (Rasch); mean ± SD					
Left foot	2.5 ± 1.8	2.5 ± 1.7	2.5 ± 1.8	2.4 ± 2.0	2.1 ± 2.3
Right foot	2.5 ± 1.9	2.6 ± 1.7	2.5 ± 1.9	2.5 ± 2.0	2.3 ± 2.0
Radiographic foot OA (one or more foot joints)	255 (63.7)	90 (59.6)	79 (62.2)	68 (70.1)	18 (72.0)
Symptomatic radiographic foot OA phenotypes					
First MTP joint OA	84 (20.7)	34 (21.8)	24 (17.3)	19 (18.3)	7 (26.9)
Polyarticular OA	62 (15.3)	14 (9.0)	25 (18.0)	21 (20.2)	2 (7.7)
Hallux valgus (either foot)	189 (44.5)	57 (36.5)	59 (42.4)	62 (59.6)	11 (42.3)
Gout	15 (3.5)	7 (4.5)	3 (2.2)	3 (2.9)	2 (7.7)
Inflammatory arthritis (RA, PsA, other)	17 (4.0)	2 (1.3)	8 (5.8)	6 (5.8)	1 (3.8)

* Values are the number (%) unless indicated otherwise. BMI = body mass index; FPI = Foot Posture Index; HADS = Hospital Anxiety and Depression Scale (higher scores indicate more anxiety and depression); LCGA = latent class growth analysis; MFPDI = Manchester Foot Pain and Disability Index (higher scores indicate more pain and disability); MCS = mental component score; MTP = metatarsophalangeal; NRS = numerical rating scale; OA = osteoarthritis; PCS = physical component score; PsA = psoriatic arthritis; RA = rheumatoid arthritis; SF‐12 = Short Form 12 health survey (higher scores indicate better physical and mental health and physical function).

A 4‐trajectory model was selected as optimal based on low AIC, BIC, and SSA‐BIC values and significance of bootstrap LRT while maintaining high entropy and average posterior probabilities (Supplementary Table 1, available on the *Arthritis Care & Research* website at http://onlinelibrary.wiley.com/doi/10.1002/acr.24823). Additionally, this model identified a small but clinically important subgroup of patients reporting high pain severity.

**Figure 1:**
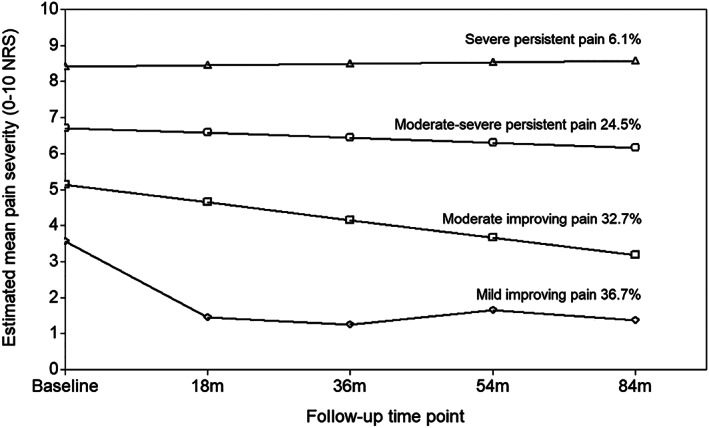
Foot pain severity trajectories over 7 years. NRS = numerical rating scale.

**Figure 2:**
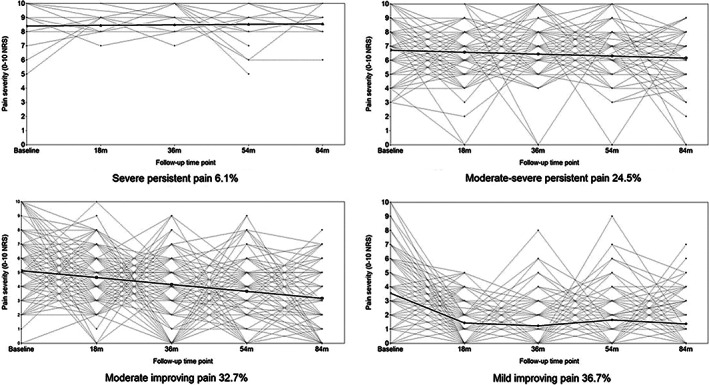
Estimated trajectories and individual observed growth trajectories. NRS = numerical rating scale.

The 4 identified trajectories were labeled as “mild, improving pain” (n = 156, 36.7%); “moderate, improving pain” (n = 139, 32.7%); “moderate‐severe, persistent pain” (n = 104, 24.5%); and “severe, persistent pain” (n = 26, 6.1%). Individuals in the mild improving pain trajectory had a mean NRS pain severity score of 3.6 at baseline that improved over 18 months and was sustained over follow‐up (slope parameter estimate = −3.513; *P* < 0.001) (Figure 1). Those in the moderate improving pain trajectory had a mean pain score of 5.1 at baseline that steadily declined over follow‐up (slope parameter estimate = −0.489; *P* < 0.001). The moderate‐severe persistent pain trajectory had a mean pain score of 6.7 at baseline that was sustained over follow‐up (slope parameter estimate = −0.138; *P* = 0.321). Finally, those in the severe persistent pain trajectory had a mean pain score of 8.4 at baseline with consistently high levels of pain over follow‐up (slope parameter estimate = 0.037; *P* = 0.787). Figure 2 depicts individual observed growth trajectories within each trajectory, and Table 1 displays the baseline characteristics for each of the 4 trajectories.

Comparable trajectories for NRS foot pain severity were obtained on complete case analysis (n = 273) (Supplementary Figure 1, available on the *Arthritis Care & Research* website at http://onlinelibrary.wiley.com/doi/10.1002/acr.24823). Similar trajectories were also identified for the MFPDI pain (n = 379) and functional limitation (n = 384) subscales scores (Supplementary Figures 2 and 3, available at http://onlinelibrary.wiley.com/doi/10.1002/acr.24823). On fitting a GMM, an optimal model with fewer trajectories (a total of 3) was identified via LCGA (Supplementary Table 2, available at http://onlinelibrary.wiley.com/doi/10.1002/acr.24823); however, this was expected because GMM allows for within‐trajectory growth variation. It was possible to replicate the 4‐trajectory model; however, the severe persistent category consisted of <4% of participants.

The baseline characteristics of individuals in the moderate‐severe and severe persistent foot pain severity trajectories (n = 130, 30.6%) were compared with those in the mild and moderate improving trajectories (n = 295, 69.4%) (Table 2). On adjustment, the less favorable long‐term foot pain NRS trajectories were significantly associated with higher BMI (adjusted OR 1.06 [95% CI 1.02–1.11]), intermediate occupations (adjusted OR 2.69 [95% CI 1.39–5.21]), routine and manual occupations (adjusted OR 2.01 [95% CI 1.15–3.53]) versus managerial and professional occupations, worse MFPDI foot pain (adjusted OR 2.19 [95% CI 1.80–2.66]) and functional limitation (adjusted OR 1.78 [95% CI 1.54–2.05]), hallux valgus in either foot (adjusted OR 2.22 [95% CI 1.43–3.44]), poorer physical (adjusted OR 0.93 [95% CI 0.91–0.95]) and mental health (adjusted OR 0.95 [95% CI 0.93–0.97]), greater anxiety (adjusted OR 1.15 [95% CI 1.09–1.21]) and depression (adjusted OR 1.21 [95% CI 1.14–1.29), and catastrophizing beliefs (adjusted OR 1.36 [95% CI 1.23–1.50]). Less favorable trajectories were not significantly associated with the presence of radiographic foot OA phenotype (first MTP joint OA adjusted OR 1.21 [95% CI 0.70–2.08] or polyarticular OA adjusted OR 1.38 [95% CI 0.74–2.54] versus no/minimal OA).

**Table 2:** Baseline characteristics and their association with moderate‐severe and severe persistent foot pain over 7 years*

Baseline characteristics	Mild and moderate improving pain (n = 295)	Moderate‐severe and severe persistent pain (n = 130)	Univariable crude OR(95% CI)	OR (95% CI) adjusted for age, sex, and BMI
Age, mean ± SD years	64.3 ± 7.7	64.5 ± 8.0	1.00 (0.98–1.03)†	1.00 (0.98–1.03)†
Sex				
Male	139 (46.1)	62 (47.7)	1	1
Female	156 (52.9)	68 (52.3)	0.98 (0.65–1.48)	0.96 (0.63–1.45)
BMI; mean ± SD	28.1 ± 4.9	29.7 ± 5.2	1.06 (1.02–1.11)†	1.06 (1.02–1.11)†
Attended higher education				
No	198 (67.1)	101 (77.7)	1	1
Yes	88 (29.8)	26 (20.0)	0.58 (0.35–0.95)	0.61 (0.37–1.01)
Occupational class				
Managerial and professional	90 (30.5)	21 (16.2)	1	1
Intermediate	51 (17.3)	31 (23.9)	2.61 (1.36–5.00)	2.69 (1.39–5.21)
Routine and manual	142 (48.1)	68 (52.3)	2.05 (1.18–3.58)	2.01 (1.15–3.53)
FPI (Rasch); mean ± SD				
Left foot	2.48 ± 1.75	2.38 ± 2.07	0.97 (0.87–1.09)†	0.97 (0.86–1.08)†
Right foot	2.55 ± 1.79	2.48 ± 2.00	0.98 (0.88–1.10)†	0.98 (0.87–1.10)†
Hallux valgus (either foot)				
Absence	179 (60.7)	57 (43.9)	1	1
Presence	116 (39.3)	73 (56.2)	1.98 (1.30–3.00)	2.22 (1.43–3.44)
MFPDI pain subscale (Rasch); mean ± SD	−0.67 ± 1.44	0.82 ± 1.23	2.23 (1.84–2.70)†	2.19 (1.80–2.66)†
MFPDI function subscale (Rasch); mean ± SD	−1.38 ± 1.86	0.63 ± 1.84	1.76 (1.54–2.01)†	1.78 (1.54–2.05)†
Radiographic foot OA phenotype				
No/minimal OA	186 (63.1)	73 (56.2)	1	1
First MTP joint OA	58 (19.7)	26 (20.0)	1.14 (0.67–1.95)	1.21 (0.70–2.08)
Polyarticular OA	39 (13.2)	23 (17.7)	1.50 (0.84–2.69)	1.38 (0.74–2.54)
SF‐12 PCS (0–100); mean ± SD	41.7 ± 11.60	32.3 ± 10.5	0.93 (0.91–0.95)†	0.93 (0.91–0.95)†
SF‐12 MCS (0–100); mean ± SD	51.7 ± 9.68	45.6 ± 11.4	0.95 (0.93–0.97)†	0.95 (0.93–0.97)†
HADS anxiety (0–21); mean ± SD	6.1 ± 3.94	8.6 ± 4.58	1.15 (1.09–1.21)†	1.15 (1.09–1.21)†
HADS depression (0–21); mean ± SD	4.4 ± 3.26	7.3 ± 4.23	1.23 (1.15–1.30)†	1.21 (1.14–1.29)†
Catastrophizing beliefs (0–6); median (IQR)‡	0 (0–2)	3 (0–5)	1.37 (1.25–1.51)†	1.36 (1.23–1.50)†

* Values are the number (%) unless indicated otherwise. 95% CI = 95% confidence interval; BMI = body mass index; FPI = Foot Posture Index; HADS = Hospital Anxiety and Depression Scale (higher scores indicate more anxiety and depression); IQR = interquartile range; MCS = mental component score; MFPDI = Manchester Foot Pain and Disability Index (higher scores indicate more pain and disability); MTP = metatarsophalangeal; OA = osteoarthritis; OR = odds ratio; PCS = physical component score; SF‐12 = Short Form 12 health survey (higher scores indicate better physical and mental health and physical function).

† Per unit increase.

‡ For catastrophizing beliefs, higher scores indicate more frequent occurrence.

Moderate‐severe and severe persistent foot pain NRS trajectories over 7 years were also accompanied by greater foot‐specific functional limitation (MFPDI function subscale) at 7 years (adjusted OR 1.43 [95% CI 1.16–1.76]) after adjustment for baseline score, age, sex, and BMI compared with those with mild and moderate improving pain trajectories.

## DISCUSSION

In community‐dwelling individuals ages ≥50 years with foot pain, we identified 4 distinct trajectories of foot pain severity over 7 years: mild improving, moderate improving, moderate‐severe persistent, and severe persistent pain. Individuals in the less favorable moderate‐severe and severe persistent pain trajectories were more likely at baseline to be obese; to have greater foot‐specific functional limitation, hallux valgus in either foot, routine/manual and intermediate occupations, and poorer physical health and mental health; and to catastrophize about their foot pain symptoms compared with those in the mild and moderate improving trajectories.

Our analysis shows that foot pain does not persist at the same intensity or progress in all individuals. Approximately one‐third of the people who reported foot pain in the last year at baseline had persistent pain over 7 years, and there was a small but important group for whom pain was persistently severe. This group consisted of 6% of the study population, and the majority (69.2%, 18 of 26) had radiographic OA, 2 of whom had concomitant gout and 1 of whom had inflammatory arthritis. On average, people in this severe persistent pain trajectory reported pain scores of 8–9 or 10 at each time point, a pain level that they are likely to consider as being unmanageable (29). In a previous community‐based study from the same geographic area, foot pain was found to persist over 3 years in more than two‐thirds of participants (30); however, persistence was defined by presence rather than severity of pain.

Although to our knowledge, foot symptom trajectories have not previously been investigated, symptom trajectories at other sites including the knee and hip have been explored (8, 9, 10, 11, 12, 31, 32, 33, 34, 35, 36, 37, 38). Studies of symptom trajectories are heterogeneous, varying by length (18 months to 14 years) and frequency (3 months to 2 years) of follow‐up (8, 32, 33), sample size (n = 222–4,796) (8, 34), and how OA was defined (8, 9, 10, 11, 32, 34), as well as age and sex characteristics of the study populations. Despite these variations, these studies found pain trajectories similar in number (between 3 and 6) and pattern (35). Trajectories are often stable, with a similar level of symptoms over time but at different thresholds (e.g., mild, moderate, and severe) (9, 10, 11, 31, 34, 35). We also found improving pain trajectories that have been similarly reported at the knee and hip (8, 12, 36, 37, 38). However, we did not identify a group of people with highly progressive pain noted in several previous pain severity studies (8, 12, 37). Although rapidly progressing forms of hip and knee OA have been established (39, 40), our results provide no indication that this occurs at the foot. Structural progression of radiographic first MTP joint OA has been reported over 3 and 19 years in approximately one‐third of participants in 2 prospective cohorts (6, 7). However, because symptoms and radiographic change are often discordant, it cannot be assumed that the individuals with persistent pain in our study also have structural progression.

Obesity, routine/manual and intermediate occupations, foot‐specific functional limitation, poorer physical and mental health, and hallux valgus have previously been found to associate with foot pain in cross‐sectional studies (41, 42, 43, 44), but few have been examined longitudinally. BMI has been found to be a predictor of future foot pain, and an increase in BMI is associated with an increase in foot pain over 2 years (45, 46) and its persistence over 6 years (47). Worsening hallux valgus severity has been associated with greater foot pain over 6 years (48, 49). Depression has been found to be a predictor of future foot pain, and poorer mental health is associated with worsening of foot pain over 3 years (45, 50). Although not examined previously at the foot, having poorer general health, poorer coping strategies, and occupation have been reported to be associated with poorer hip and knee pain trajectories (9, 12, 36).

Pain has previously been found to be associated with increased risk of functional impairment development and progression, with stronger associations found for increased pain severity (51, 52, 53). The mechanisms underlying the association between pain and functional decline are not fully understood, but it is thought that individuals with joint pain often become less active often to avoid the pain and/or believe that they could make their condition worse (54). It is also believed that pain can reduce joint range of motion (55). In the long term, both these pathways are considered to result in the deterioration of physical performance and muscle weakness, which gradually lead to functional impairment and disability (56).

The strengths of this study include a prospective cohort design in a community‐dwelling population not selected based on primary care consultation or referral. It is the first study, to our knowledge, to examine trajectories of foot pain prospectively over several follow‐up time points. The quality of classification of the 4‐trajectory model was high, as judged by the average posterior probabilities and entropy value. The model was robust to missing data and model assumptions. Furthermore, similar trajectories were replicated in the complete case analysis, despite evidence of attrition bias, and for other foot outcomes.

A number of limitations should also be acknowledged. The source study population, the CASF study, has low representation of ethnic minorities (13). Pain severity was collected at the person level and so included both feet. It is therefore possible that the specific foot or regions of the foot with the most severe pain varied over time. Incident cases of foot pain were not specifically examined, and it is possible that the different trajectories may represent different stages of foot disease. Individuals with OA commonly report fluctuations and flares in their symptoms, and it is unknown how these short‐term changes are reflected in the trajectories because the interval between data collection points was fairly long (57, 58). Equidistance between follow‐up time points was assumed in the analysis, which was the case for 4 out of 5 time points. The time period between the penultimate and last time points was 6 months longer, but this is unlikely to have had an effect on the results over 7 years. It is unknown to what extent clinical treatment and self‐management influenced the trajectories identified. However, previous studies suggest that only a minority of individuals with foot pain consult primary care, with most prescribed analgesics and few receiving onward treatment referrals (59, 60). Additionally, some of the baseline characteristics in the nested case–control analysis, such as the symptomatic radiographic phenotypes, were less prevalent than anticipated, leading to insufficient power and a type II error.

Our study has important implications for individuals with foot pain, clinicians, and policy makers. Individuals with joint pain often believe that it is inevitable that their pain will get worse over time (61, 62); however, our findings provide reassurance that not all foot pain is progressive, and many people will experience some improvement over time. Our findings will aid in identification of individuals with moderate and severe persistent pain at risk of worse outcomes who could be targeted for intervention. Hallux valgus and obesity were associated with progressive pain trajectories, and each are potentially modifiable with orthoses and corrective surgery for hallux valgus and with diet and exercise for obesity (63, 64, 65). There is a need for well‐designed intervention studies to determine whether individuals with different trajectories would benefit from early intervention and whether specific interventions for hallux valgus and obesity influence foot symptoms.

In conclusion, over a 7‐year period, one‐third of individuals had persistent moderate to severe or severe pain that was accompanied by a decline in foot‐specific function. Obesity and hallux valgus have been identified as possible prognostic factors. Further investigation to ascertain whether interventions targeting these factors improve long‐term outcomes in people with foot pain are warranted.

## AUTHOR CONTRIBUTIONS

All authors were involved in drafting the article or revising it critically for important intellectual content, and all authors approved the final version to be submitted for publication. Dr. Marshall had full access to all of the data in the study and takes responsibility for the integrity of the data and the accuracy of the data analysis.

### Study conception and design

Marshall, Blagojevic‐Bucknall, Rathod‐Mistry, Thomas, Edwards, Peat, Menz, Roddy.

### Acquisition of data

Marshall, Thomas, Roddy.

### Analysis and interpretation of data

Marshall, Blagojevic‐Bucknall, Rathod‐Mistry, Thomas, Edwards, Peat, Menz, Roddy.

## Supporting information


Disclosure Form

**Appendix S1** Supplementary Information
