# Supplementary material for: Identifying Long‐Term Trajectories of Foot Pain Severity and Potential Prognostic Factors: A Population‐Based Cohort Study
Source: Arthritis Care Res (Hoboken). 2022 Nov 25;75(5):1123–31. doi: 10.1002/acr.24823 (PMC10952181; doi:10.1002/acr.24823)
Supplement: Supplementary file 2 — Appendix S1 Supplementary Information [file ACR-75-1123-s001.docx]

**Supplementary Table 1. LCGA goodness of fit measures for mixed, linear, quadratic and cubic polynomial trajectories**

| **Number of trajectories** | **AIC** | **BIC** | **SSA-BIC** | **LMR-LRT** | **B-LRT** | **Entropy** | **Average posterior probabilities** | **Trajectory membership proportions** |
| --- | --- | --- | --- | --- | --- | --- | --- | --- |
| **Mixed polynomial model** | | | | | | | | |
| 1 * | 9418.14 | 9454.61 | 9426.05 | NA | NA | NA | 1.0 | 1.0 |
| 2 † | 8817.19 | 8865.81 | 8827.73 | p<0.001 | p<0.001 | 0.818 | 0.943, 0.953 | 0.41, 0.59 |
| 3 ‡ | 8701.97 | 8762.75 | 8715.15 | p=0.0003 | p<0.001 | 0.756 | 0.844, 0.893, 0.925 | 0.39, 0.22, 0.39 |
| **4 §** | **8690.17** | **8763.11** | **8705.99** | **p=0.1828** | **p<0.001** | **0.723** | **0.779, 0.841, 0.808, 0.912** | **0.24, 0.06, 0.33, 0.37** |
| 5 Ⱡ | 8656.29 | 8745.44 | 8675.62 | p=0.2575 | p<0.001 | 0.705 | 0.763, 0.860, 0.763, 0.749, 0.856 | 0.23, 0.15, 0.20, 0.11, 0.31 |
| **Linear polynomial model** | | | | | | | | |
| 1 | 9432.19 | 9460.55 | 9438.34 | NA | NA | NA | 1.0 | 1.0 |
| 2 | 8862.28 | 8902.80 | 8871.07 | p<0.001 | p<0.001 | 0.802 | 0.946, 0.939 | 0.59, 0.41 |
| 3 | 8754.64 | 8807.32 | 8766.06 | p=0.0004 | p<0.001 | 0.732 | 0.907, 0.828, 0.895 | 0.39, 0.40, 0.21 |
| 4 | 8743.99 | 8808.82 | 8758.05 | p=0.1345 | p<0.001 | 0.688 | 0.840, 0.779, 0.774, 0.874 | 0.06, 0.35, 0.25, 0.34 |
| 5 | 8729.36 | 8806.35 | 8746.06 | p=0.3393 | p<0.001 | 0.689 | 0.789, 0.714, 0.737, 0.852, 0.849 | 0.24, 0.15, 0.19, 0.28, 0.14 |
| **Quadratic polynomial model** | | | | | | | | |
| 1 | 9424.88 | 9457.30 | 9431.91 | NA | NA | NA | 1.0 | 1.0 |
| 2 | 8833.67 | 8882.29 | 8844.21 | p=0.0124 | p<0.001 | 0.814 | 0.933, 0.956 | 0.42, 0.58 |
| 3 | 8721.93 | 8786.76 | 8735.99 | p=0.0010 | p<0.001 | 0.747 | 0.845, 0.917, 0.888 | 0.38, 0.39, 0.23 |
| 4 | 8704.06 | 8785.10 | 8721.63 | p=0.5761 | p<0.001 | 0.745 | 0.818, 0.905, 0.890, 0.716 | 0.32, 0.35, 0.23, 0.10 |
| 5 | 8662.75 | 8759.99 | 8683.84 | p=0.1233 | p<0.001 | 0.725 | 0.763, 0.779, 0.777, 0.872, 0.854 | 0.16, 0.21, 0.19, 0.30, 0.14 |
| **Cubic polynomial model** | | | | | | | | |
| 1 | 9418.14 | 9454.61 | 9426.05 | NA | NA | NA | 1.0 | 1.0 |
| 2 | 8819.57 | 8876.30 | 8831.87 | p<0.001 | p<0.001 | 0.818 | 0.940, 0.955 | 0.42, 0.58 |
| 3 | 8705.75 | 8782.74 | 8722.45 | p=0.0011 | p<0.001 | 0.754 | 0.903, 0.844, 0.920 | 0.23, 0.39, 0.38 |
| 4 | 8688.43 | 8785.68 | 8709.52 | p=0.2696 | p<0.001 | 0.748 | 0.795, 0.915, 0.887, 0.754 | 0.30, 0.36, 024, 0.10 |
| 5 | 8650.57 | 8768.08 | 8676.05 | p=0.3334 | p<0.001 | 0.719 | 0.770, 0.774, 0.870, 0.854, 0.760 | 0.21, 0.16, 0.29, 0.14, 0.20 |

*AIC Akaike Information Criterion; BIC Bayesian Information Criterion; SSA-BIC sample size adjusted BIC; LRT Likelihood ratio test; LMR-LRT Lo, Mendell and Rubin adjusted LRT; B-LRT Bootstrap LRT.*T1:Cubic; †T1: Linear, T2: Cubic; ‡T1 Linear, T2: Linear, T3: Cubic; §T1: Linear, T2: Linear, T3: Linear, T4:Cubic; ⱠT1: Linear, T2: Linear, T3: Linear, T4: Quadratic, T5: Cubic. The bold text indicates the model that was selected as having the optimal number or classes.*

**Supplementary Figure 1. Sensitivity analysis: Foot pain severity trajectories over seven years for complete-case analysis (pain severity (0-10 NRS) data required at all five time-points)**


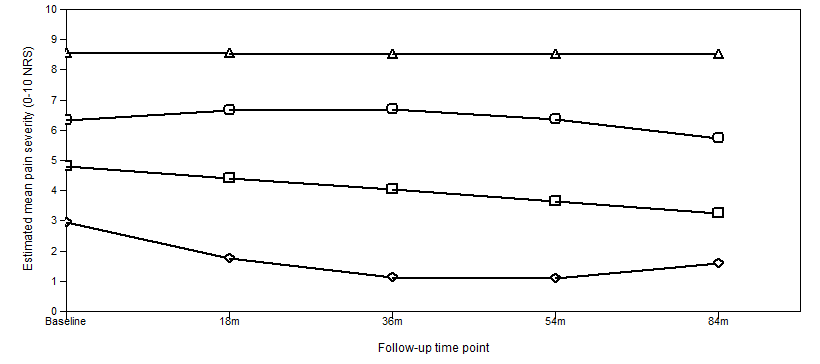


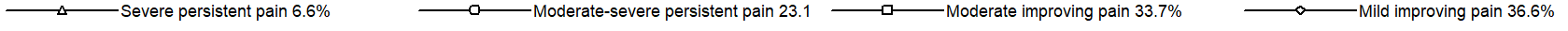


**Supplementary Figure 2. Sensitivity analysis: MFPDI pain subscale trajectories over seven years; data at baseline and at least two follow-up time points required**


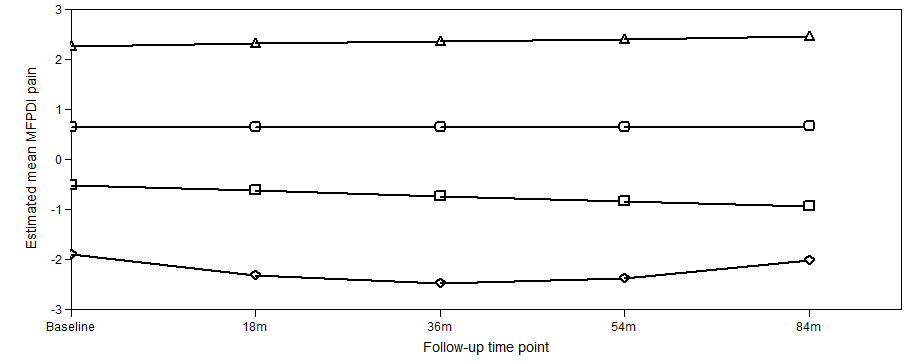


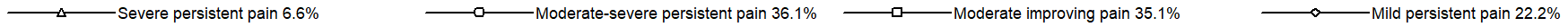


**Supplementary Figure 3. Sensitivity analysis: MFPDI function subscale trajectories over seven years; data at baseline and at least two follow-up time points required**


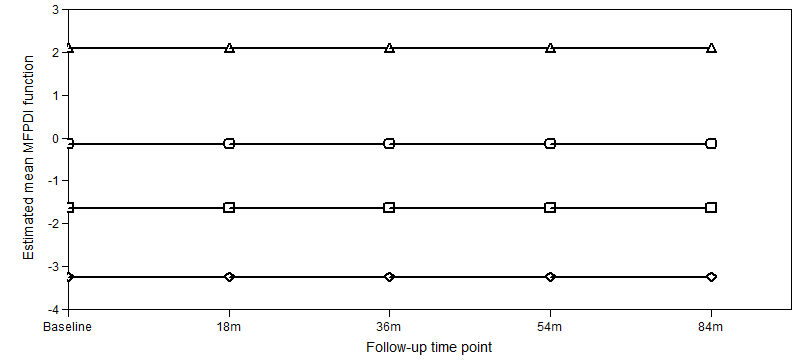


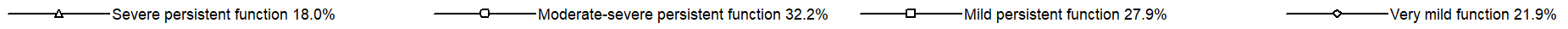


**Supplementary Table 2. Sensitivity analysis: GMM goodness of fit measures for mixed polynomial model for pain severity (0-10 NRS); data at baseline and at least two follow-up time points required**

| **Number of trajectories** | **AIC** | **BIC** | **SSA-BIC** | **LMR-LRT** | **B-LRT** | **Entropy** | **Average posterior probabilities** | **Trajectory membership proportions** |
| --- | --- | --- | --- | --- | --- | --- | --- | --- |
| 1 | 8720.01 | 8780.79 | 8733.19 | na | na | na | 1.0 | 1.0 |
| 2 | 8659.43 | 8756.68 | 8680.52 | p=0.0458 | p<0.001 | 0.851 | 0.982, 0.785 | 0.85, 0.15 |
| 3 | 8652.00 | 8745.20 | 8672.21 | p=0.0980 | p<0.001 | 0.680 | 0.895, 0.770, 0.797 | 0.60, 0.18, 0.22 |
| 4 | 8648.81 | 8754.16 | 8671.65 | p=0.0593 | p<0.001 | 0.734 | 0.782, 0.823, 0.900, 0.717 | 0.20, 0.01, 0.63, 0.16 |

*AIC Akaike Information Criterion; BIC Bayesian Information Criterion; SSA-BIC sample size adjusted BIC; LRT Likelihood ratio test; LMR-LRT Lo, Mendell and Rubin adjusted LRT; B-LRT Bootstrap LRT.*

Notes: In LCGA, within-trajectory variances (i.e. variances of intercepts, slopes etc.) are set to zero, thereby assuming homogeneity of individual trajectory growth in each estimated trajectory. In GMM, these variances are freely estimated. Examination of Figure 2 in the main manuscript suggests that it is reasonable to allow variances of all corresponding growth parameters (i.e. intercepts, slopes etc) to vary within each trajectory. Same model selection procedure is used as in LCGA. Where MPlus output indicated negative estimated variances, residual variances for the slope were examined; if small and insignificant, they were set to zero for the corresponding trajectory. No model improvement in model fit was seen when considering linear, quadratic or cubic only polynomial formulations for each trajectory.
